# Optimizing planting times and weed management for enhanced yield and nutritional quality of baby corn and green fodder

**DOI:** 10.1371/journal.pone.0308296

**Published:** 2024-09-06

**Authors:** Md. Rejaul Haque, Md. Abdur Rahman Sarkar, Md. Sojib Kabiraj, Md. Abdur Razzak, Shubroto Kumar Sarkar, Md. Harun Rashid, Swapan Kumar Paul

**Affiliations:** 1 Department of Agronomy, Bangladesh Agricultural University, Mymensingh, Bangladesh; 2 Department of Crop Botany, Bangladesh Agricultural University, Mymensingh, Bangladesh; University of Agriculture, PAKISTAN

## Abstract

A study was carried out to ascertain the impact of planting time and weeding schedule on the yield and quality of baby corn. The trial included three planting times viz. 15 November, 15 December and 14 January and five levels of weeding regime viz. no weeding, two hand weeding (HW) at 15 days after sowing (DAS) and 30 DAS, herbicide pendimethalin @ 2.5 L ha^-1^ (pre-emergence) + one HW at 30 DAS, herbicide pyrazosulfuran-ethyl @ 2.0 L ha^-1^ (post-emergence) + one HW at 30 DAS and combined herbicide of pendimethalin + pyrazosulfuran-ethyl. The maximum cob yield with husk (11.93 t ha^-1^) and cob yield without husk (3.07 t ha^-1^) were obtained from the 15 December sowing with the application of pendimethalin followed by pyrazosulfuran-ethyl. Plants sown on 15 December with the application pyrazosulfuran-ethyl with one HW at 30 DAS gave the highest protein content in cobs (20.20%), while the leaf protein content showed the highest result (18.70%) in the plants sown on 15 December with no weeding. Therefore, it can be concluded that the baby corn might be planted on December 15 with the combined application of pendimethalin + pyrazosulfuran-ethyl herbicides and pyrazosulfuran-ethyl + one HW at 30 DAS for maximum cob yield and protein content, respectively.

## Introduction

Grains make up a larger portion of all crops used to produce most of the food consumed by humans. To feed the world’s rising population, one of the key goals is to increase agricultural productivity. Maize ranks as the third most significant cereal crop globally following wheat and rice in importance. This plant serves multiple purposes when cultivated such as supplying food, fuel and raw materials for some industrial items [1,2]. Both tropical and chilly climes benefit from it. It may thrive in a wide range of agroclimatic settings. Baby corn (*Zea mays* L.) is defined as juvenile corn cobs that have been harvested whole and entirely edible at the silk emergence stage (2–3 cm long) just before fertilization [3]. It is incredibly palatable, sweet, and easy to consume because of its softness and sweetness with high nutritional value [4]. The immature cob is firmly wrapped within the husk and well protected from insects and pests when harvested, resulting in a crop that is not only nutritious but also free of pesticide residues [5]. Because of people’s shifting dietary preferences, short growing season (70–80 days), and high export potentiality baby corn production is becoming popular in Bangladesh [6]. The expansion of the food processing sector has led to an increase in demand for baby corn on a global scale [7,8]. Given its short growing season, it is a crop that is readily incorporated into an intensive cropping system which is a great way to support Bangladesh’s economy and fight against poverty.

Suitable planting times and proper weed management are crucial factors for the successful cultivation of any crop. Crop growth, development, yield and yield components are all significantly impacted by the planting date [9–12]. By planting hybrids with various maturation dates at various times, producers can extend their harvest [13]. In general, early seed sowing lengthens the maturation process, whereas late seed sowing accelerates it and reduces yield [14]. Furthermore, planting crops at the right time of year encourages weed emergence when weed populations are at their lowest. According to Nurse and DiTommaso [15], variations in the growth environment can also affect the quantity, composition, viability and capacity of seeds to germinate. The first 30 to 60 days following planting in maize are regarded as a crucial time for crop weed competition [16]. According to crop-weed competition intensity and duration, yield loss caused by weeds in maize ranges from 28 to 93% [17]. It also depends on the kind of weed flora. According to Pandey et al. [18], season-long weed infestation decreased the production of baby corn by 44.0%, while [19] estimated that weeds are responsible for roughly 37% of the yield losses in maize globally. Hand weeding may occasionally be challenging, labor-intensive, expensive and time-consuming yet incredibly effective in managing weeds. On the other hand, herbicide treatment gives stronger establishment and competitive abilities [20]. Since the invention of herbicides, controlling weeds in maize has become easier and inexpensive. There are just a few herbicides that can be used to manage weeds in maize, including atrazine, pendimethalin, metribuzin, and 2, 4-D [21]. Weeds in maize are being controlled by cultural and chemical means. Adjusting planting times to align with optimal climatic conditions and implementing targeted weed management practices that will reduce competition for resources leading to higher productivity and better nutritional quality of the baby corn and green fodder. Therefore, the study was undertaken to find out the optimum time of sowing and better weed control option for maximizing yield and nutritional quality of baby corn and green fodder from the same crop of maize.

## Materials and methods

### Experimental site

**Fig 1:**
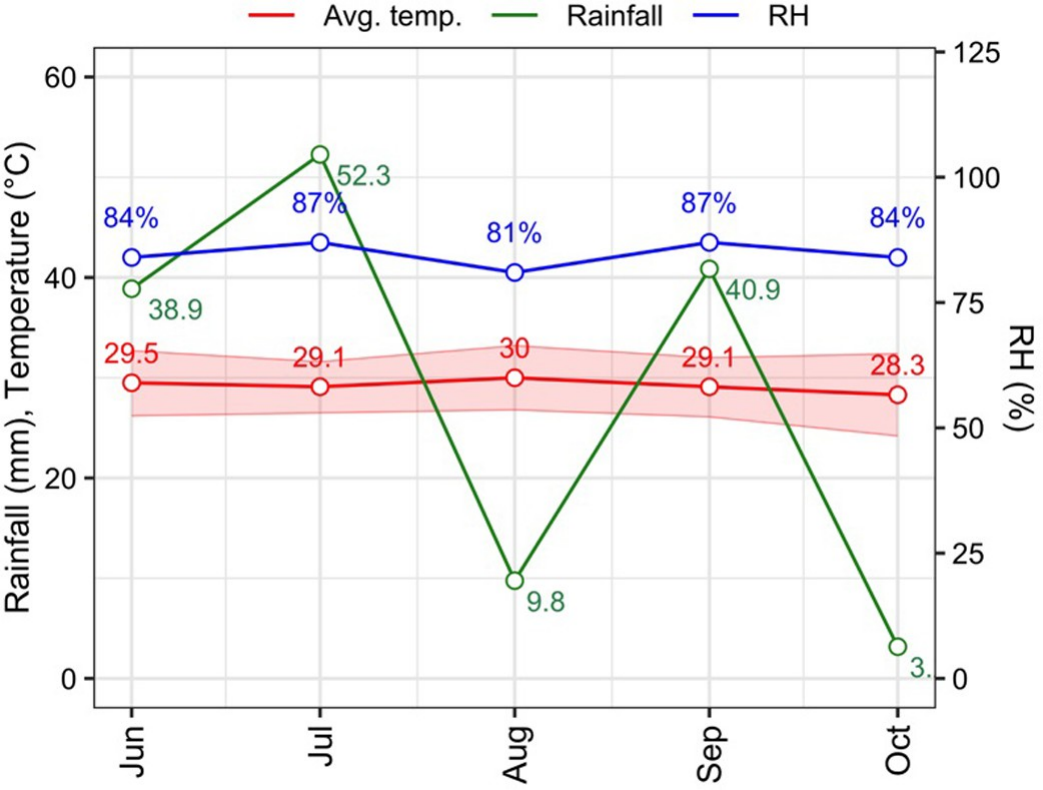
The average temperature, rainfall and relative humidity in crop growing period.

The trial was conducted at the Agronomy Field Laboratory, Bangladesh Agricultural University in Mymensingh from November 2016 to May 2017. The location of the site was at 24°25’ N latitude and 90°50’ E longitude with an elevation of 18 meters which is characterized by non-calcareous dark grey floodplain soil within the Agroecological Zone (AEZ-9) of the Old Brahmaputra Floodplain [22]. Climatic data such as temperature, relative humidity, precipitation and sunshine hours were depicted in Fig 1.

### Experimental treatments

The study included three dates of planting viz. 15 November (D_1_), 15 December (D_2_) and 14 January (D_3_) and five weed managements viz. no weeding (W_0_), two hand weeding (HW) at 15 DAS and 30 DAS (W_1_), herbicide pendimethalin @ 2.5 L ha^-1^ (pre-emergence) + one HW at 30 DAS (W_2_), herbicide pyrazosulfuran-ethyl @ 2.0 L ha^-1^ (post-emergence) + one HW at 30 DAS (W_3_) and combine herbicide of pendimethalin + pyrazosulfuran-ethyl (W_4_). The experiment was set up following a randomized complete block design (RCBD) which was replicated thrice (S1 Fig).

### Plant management

The land was organized thoroughly with tilling twice by a power tiller followed by laddering. Individual plots were cleaned by removing weeds and properly leveled. Different fertilizers were applied recommended at urea, triple super phosphate, muriate of potash, gypsum, zinc sulphate and boric acid @ 250 kg, 150 kg, 100 kg, 75 kg, 10 kg, and 5 kg ha^-1^. All fertilizers except urea were administered during the final stages of land preparation. Urea was divided into two halves with half applied during land preparation and the remaining half distributed equally in two portions at 30 and 60 DAS. Two baby corn (Baby Star) seeds were dribbled in a hill in furrows minimizing the missing or failure of germination hill^-1^. Seeds were placed in each point at 4–5 cm depth from the soil surface with 45 cm × 20 cm spacing. After sowing, seeds were covered with a thin layer of soil. Intercultural activities like thinning, earthing up, watering and pest control were performed as needed.

### Data collection

The weed flora encompassing grasses, broadleaf weeds, and sedges was populated by ten weed species from seven different families (Table 1). Most of these weed species were annual plants. We gathered data on weed species by employing a 1.0 m × 1.0 m quadrat following the method outlined by Cruz et al. [23] at the harvest stage in each plot. The quadrat was positioned randomly in three locations within each plot and then converted to a density per square meter. After tallying the weed density, the weeds within each quadrat were removed, cleaned, sorted by species and dried in an electric oven for 72 hours at 80°C. Young cobs were harvested 2–3 days after silking when the silk length measured 3–4 cm and were handpicked during harvesting. During harvesting, five plants were chosen at random to collect data on yield components.

**Table 1:** Infesting weed species in the experimental plots of baby corn.

Sl no.	Local name	Scientific name	Family	Life cycle
1	Biskatali	*Polygonum hydropiper* L.	Polygonaceae	Annual
2	Bathua	*Chenopodium album* L.	Amaranthaceae	Annual
3	Mutha	*Cyperous rotundus* L.	Cyperaceae	Annual
4	Foskabegun	*Physalis heterophylla* L.	Solanaceae	Perennial
5	Durba	*Cynodon dactylon* L.	Gramineae	Annual
6	Tithbegun	*Solanum torvum* L.	Solanaceae	Perennial
7	Chapra	*Elusine indica* L.	Poaceae	Annual
8	Shama	*Echinochloa cruss-galli* L.	Gramineae	Annual
9	Khudeshama	*Echinochloa colonum* L.	Gramineae	Annual
10	Amruli grass	*Oxalis europea* L.	Oxalidaceae	Perennial

### Chemical analysis

Cobs, leaves and stems were gathered from each plot and then dried and cleaned manually. Afterward, the dried samples were ground using a grinding mill with 60 mesh sieves. These prepared samples were stored in polythene bags within a desiccator for later chemical analysis to determine protein content. The percentage of protein was assessed using the Micro-Kjeldahl method [24]. Ground cob, leaf and stem sample amounting 0.5 g was taken in a 75 ml Kjeldahl flask along with 3 ml concentrated H_2_SO_4_. 2 ml H_2_O_2_, one Kjeldahl tablet (1.5 g K_2_SO_4_ + 0.0075 g selenium) and 2 glass balls. The sample mixture was heated at 370°C over preheated heater for 1 hour. After the completion of digestion the sample was cooled and diluted up to the mark with distilled water. Ten ml of digested diluted solution was distilled with 10 ml of 40% NaOH with the help of distillation apparatus. The distillate was collected in 10 ml 20% boric acid solution containing 2 drops of mixed indicator (0.02 g methyl red + 0.01869 methelene blue in 20 ml ethanol). The distillate was titrated with 0.01 N HCI solutions. Calculation was done by the following the formula analysis for estimation of protein:

$$\%\text{Nitrogen}=\frac{(\text{Sample}\;\text{liter}-\text{Blank}\;\text{liter})\times \text{Normality}\;\text{of}\;\text{HCl}\times 0.014\times 100}{\text{Sample}\;\text{weight}(\text{g})}$$

Here, Normality of HCI = 0.01N, 0.014 = Nitrogen factor, 100 = Percentage, Percentage of protein was obtained by multiplying % Nitrogen with the factor 5.95, % Protein = % Nitrogen × 6.25 (6.25 = Protein factor)

Total carbohydrates were assessed following the protocols outlined by Raghuramulu et al. [25].


$$\text{Carbohydrate}(\text{g}/100\text{g}\;\text{sample})=\frac{100-[(\text{moisture}+\text{fat}+\text{protein}+\text{ash}+\text{crude}\;\text{fiber})\text{g}}{100\text{g}}$$

The fat content was measured through continuous solvent extraction using a Soxhlet apparatus, as described by Hughes [26]. Fiber and ash determination followed the procedure outlined by Ranganna [27]. The level of moisture in baby corn and fodder samples was assessed following the outlined procedure by Ezeagu [28].


$$\text{Moisture}(\%)=\frac{(\text{Initial}\;\text{weight}-\text{Final}\;\text{weight})\times 100}{\text{Weight}\;\text{of}\;\text{sample}}$$

Sulfur content was assessed using colorimetric methods (measuring BaSO_4_ turbidity), while potassium, calcium and phosphorus were analyzed using an atomic absorption spectrophotometer proposed by Yoshida [29].

### Statistical analysis

Analysis of variance (ANOVA) was done to investigate the significant differences in the recorded parameters resulting from the experimental treatments. Mean differences were adjudged by Duncan’s Multiple Range Test (DMRT) [30]. Statistical analyses were performed using the software program Statistix 10.

## Results

### Effects of planting times and weeding strategies on weed infestation in baby corn

The combination of planting date and weed management strategies significantly affected both weed density and weed dry matter (TDM) production (Table 2). The uppermost weed density (271.67 m^-2^) was calculated in the plants sown on 14 January without weeding application (D_3_ × W_0_) which was at par with 15 December planting without weeding plots (D_2_ × W_0_). The minimum density of weeds (35.0 m^-2^) was recorded in the plots of 15 November planting with the application of herbicide pendimethalin @ 2.5 L ha^-1^ with one HW at 30 DAS (D_1_ × W_2_). In case of weed dry matter production, the maximum TDM (74.59 g) was recorded in the plants sown on 14 January without weeding management (D_3_ × W_0_) which was similar with both on 15 November and 15 December planting under same weeding management. The minimum TDM (2.74 g) production was found in the plants of 15 December planting with the application of herbicide pendimethalin @ 2.5 L ha^-1^ followed by pyrazosulfuran-ethyl @ 2.0 L ha^-1^ (D_2_ × W_4_) which was similar with the application of herbicide pendimethalin @ 2.5 L ha^-1^ with one HW at 30 DAS under same planting time (D_2_ × W_2_).

**Table 2:** Effect of date of sowing on weed density and total dry matter production of weeds.

Date of planting × weeding		Number of weeds m^-2^	Weed dry matter production m^-2^ (g)
15 November (D_1_)	W_0_	226 ± 34.22 b	71.64 ± 13.06 a
	W_1_	60.67 ± 10.27 cd	19.11 ± 2.55 cd
	W_2_	35 ± 6.64 e	11.46 ± 2.48 def
	W_3_	45.67 ± 8.41 de	14.98 ± 2.57 cde
	W_4_	51.33 ± 9.46 de	18.78 ± 2.56 cd
15 December (D_2_)	W_0_	254 ± 55.32 a	52.57 ± 9.58 b
	W_1_	79 ± 12.76 c	6.77 ± 1.48 fg
	W_2_	44 ± 5.81 de	4.23 ± 0.79 fg
	W_3_	39 ± 6.73 de	7.1 ± 1.15 fg
	W_4_	42 ± 5.99 de	2.74 ± 0.42 g
14 January (D_3_)	W_0_	271.67 ± 45.81 a	74.59 ± 15.18 a
	W_1_	81 ± 12.72 c	21.82 ± 2.93 c
	W_2_	36.67 ± 8.01 de	10.8 ± 1.5 ef
	W_3_	43.67 ± 6.94 de	14.98 ± 3.14 cde
	W_4_	37.67 ± 5.38 de	10.58 ± 1.6 ef
Significance level		*	***

In a column, figures with same letter (s) do not differ significantly whereas figures with dissimilar letter differ significantly (as per DMRT), *, ** and *** = Significant at 5%, 1% and 0.1% level of probability, respectively. Here, W_0_ = No weeding, W_1_ = Two hand weeding at 15 days after sowing (DAS) and 30 DAS, W_2_ = Pre-emergence herbicide (Pendimethalin) followed by one hand weeding at 30 DAS, W_3_ = Post-emergence herbicide (Pyrazosulfuran-ethyl) followed by one hand weeding at 30 DAS, W_4_ = Pre-emergence herbicide (Pendimethalin) followed by Post-emergence herbicide (Pyrazosulfuran-ethyl).

### Effects of planting times and weeding regime on yield components

The yield components were greatly impacted by both the timing of planting and the methods used for weeding (Table 3). The maximum plant height (138.48 cm) was observed in plants sown on 15 November using herbicide pendimethalin @ 2.5 L ha^-1^ with one HW at 30 DAS (D_1_ × W_2_) which was identical to planted on 15 November with applying herbicide pyrazosulfuran-ethyl @ 2.0 L ha^-1^ with one HW at 30 DAS (D_1_ × W_3_) while the lowest one (85.45 cm) was found on 15 November planting with no weeding (D_1_ × W_0_). The plant produced a maximum number of cobs (2.36 plant^-1^) when planted on 15 November applying herbicide pyrazosulfuran-ethyl @ 2.0 L ha^-1^ with one HW at 30 DAS (D_1_ × W_3_) and the minimum cobs number (0.26 plant^-1^) was counted from without weeded plots under same planting time (D_1_ × W_0_). The uppermost cob length without husk (21.51 cm), cob length without husk (10.29 cm) and cob diameter with husk (2.28 cm) were found when planted on 15 November using herbicide pendimethalin @ 2.5 L ha^-1^ and pyrazosulfuran-ethyl @ 2.0 L ha^-1^ (D_1_ × W_4_). The lowest performance (4.74 cm), (2.28 cm) and (0.49 cm) were recorded when planted on 15 November with no weeding (D_1_ × W_0_). Whereas the highest cob diameter without husk (1.45 cm) resulted on 15 December with the application of herbicide pendimethalin @ 2.5 L ha^-1^ and pyrazosulfuran-ethyl @ 2.0 L ha^-1^ (D_2_ × W_4_) and the lowest one (0.32 cm) was in shown on 15 November with no weeding (D_1_ × W_0_).

**Table 3:** Effect of interaction between date of sowing and weeding regime on crop characters and yield components of baby corn.

Planting dates × weeding		Plant height (cm)	Number of cobs plant^-1^	Cob length with husk (cm)	Cob length without husk (cm)	Cob diameter with husk (cm)	Cob diameter without husk (cm)
15 November (D_1_)	W_0_	85.45 ± 13.02 c	0.26 ± 0.04 e	4.74 ± 0.71 4d	2.28 ± 0.31 e	0.49 ± 0.08 g	0.32 ± 0.06 g
	W_1_	126.01 ± 19.08 ab	1.86 ± 0.34 c	20.61 ± 3.12 ab	9.89 ± 1.38 ab	2.03 ± 0.31 a-c	1.31 ± 0.22 a-c
	W_2_	138.48 ± 23.44 a	2.33 ± 0.31 ab	19.14 ± 3.3 ab	9.26 ± 1.71 ab	2.19 ± 0.45 ab	1.4 ± 0.24 ab
	W_3_	135.64 ± 25.74 a	2.36 ± 0.51 a	20.67 ± 2.76 ab	9.91 ± 2.16 ab	2 ± 0.27 a-c	1.29 ± 0.17 a-d
	W_4_	123.56 ± 22.75 ab	2.23 ± 0.38 a-c	21.51 ± 3.28 a	10.29 ± 1.87 a	2.28 ± 0.32 a	1.39 ± 0.26 ab
15 December (D_2_)	W_0_	110.17 ± 20.31 b	0.67 ± 0.09 de	14.22 ± 2.41 c	6.82 ± 1.43 d	1.37 ± 0.29 ef	0.92 ± 0.14 ef
	W_1_	133.58 ± 29.09 a	2 ± 0.36 a-c	19.67 ± 2.8 ab	9.44 ± 2.06 ab	1.84 ± 0.28 a-e	1.2 ± 0.21 a-e
	W_2_	124.16 ± 20.05 ab	2.06 ± 0.45 a-c	18.89 ± 4.09 ab	9.06 ± 1.44 ab	1.86 ± 0.28 a-d	1.21 ± 0.16 a-e
	W_3_	120.75 ± 15.93 ab	2.06 ± 0.38 a-c	18.78 ± 3.82 ab	9.02 ± 1.97 ab	1.66 ± 0.29 c-e	1.17 ± 0.18 a-e
	W_4_	124.42 ± 21.48 ab	2.06 ± 0.33 a-c	18.67 ± 3.54 ab	8.96 ± 1.35 a-c	2.26 ± 0.3 a	1.45 ± 0.25 5a
14 January (D_3_)	W_0_	110.5 ± 15.75 b	0.8 ± 0.12 d	14.45 ± 2.44 c	6.92 ± 1.19 cd	1.04 ± 0.16 f	0.72 ± 0.1 f
	W_1_	133.83 ± 22.57 a	2.16 ± 0.44 a-c	17.11 ± 2.93 bc	8.21 ± 1.12 b-d	1.46 ± 0.25 def	1.01 ± 0.22 d-f
	W_2_	126.83 ± 19.92 ab	1.93 ± 0.26 bc	17.56 ± 2.36 a-c	8.42 ± 1.53 a-d	1.6 ± 0.23 c-e	1.07 ± 0.22 c-e
	W_3_	121.42 ± 26.51 ab	2.03 ± 0.28 a-c	17 ± 3.13 bc	8.16 ± 1.78 b-d	1.52 ± 0.33 de	1.01 ± 0.19 d-f
	W_4_	124.67 ± 19.8 ab	2 ± 0.42 a-c	16.89 ± 2.65 bc	7.95 ± 1.49 b-d	1.72 ± 0.35 b-e	1.13 ± 0.19 b-e
Significance level		*	*	***	**	***	***

In a column, figures with same letter (s) do not differ significantly whereas figures with dissimilar letter differ significantly (as per DMRT), *, ** and *** = Significant at 5%, 1% and 0.1% level of probability, respectively. Here, W_0_ = No weeding, W_1_ = Two hand weeding at 15 days after sowing (DAS) and 30 DAS, W_2_ = Pre-emergence herbicide (Pendimethalin) followed by one hand weeding at 30 DAS, W_3_ = Post-emergence herbicide (Pyrazosulfuran-ethyl) followed by one hand weeding at 30 DAS, W_4_ = Pre-emergence herbicide (Pendimethalin) followed by Post-emergence herbicide (Pyrazosulfuran-ethyl).

### Cob yield under different planting times and weeding regime

**Fig 2:**
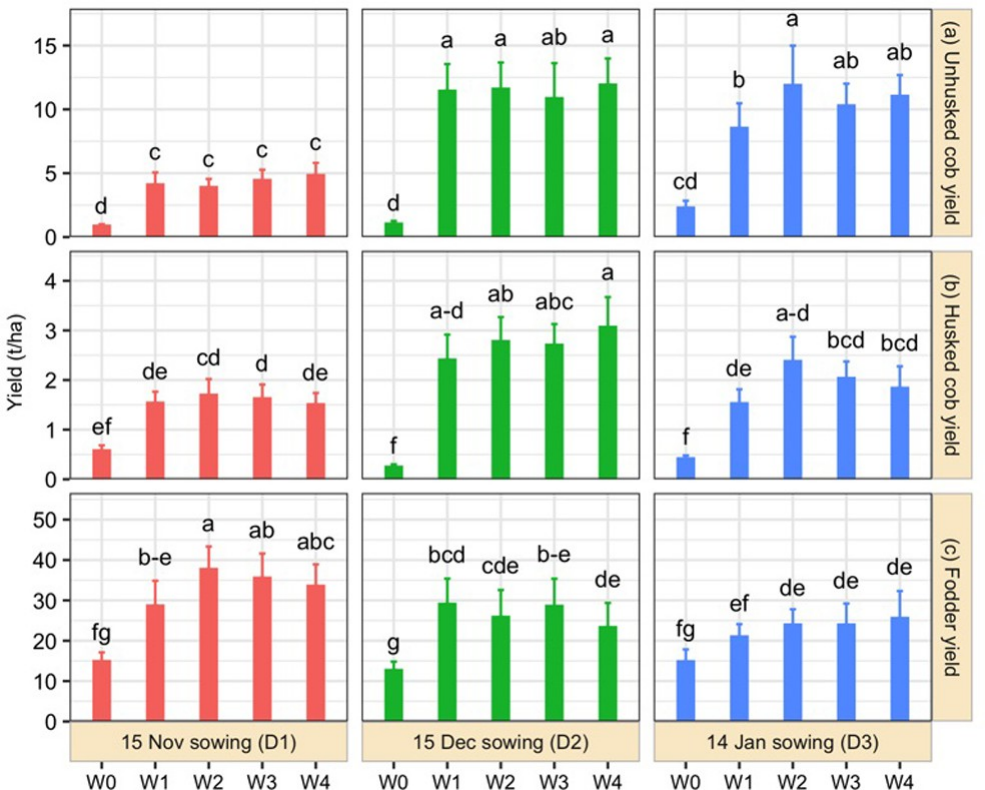
Effects of date of planting and weeding regimes on baby corn and green fodder yield. Here, W_0_ = No weeding, W_1_ = Two hand weeding at 15 days after sowing (DAS) and 30 DAS, W_2_ = Pre-emergence herbicide (Pendimethalin) followed by one hand weeding at 30 DAS, W_3_ = Post-emergence herbicide (Pyrazosulfuran-ethyl) followed by one hand weeding at 30 DAS, W_4_ = Pre-emergence herbicide (Pendimethalin) followed by Post-emergence herbicide (Pyrazosulfuran-ethyl).

The yield of the baby corn cob was significantly influenced by the interactions between the various treatments (Fig 2A and 2B). The maximum cob yield with husk (11.93 t ha^-1^) and cob yield without husk (3.07 t ha^-1^) were measured at planting on 15 December by applying herbicide pendimethalin @ 2.5 L ha^-1^ and pyrazosulfuran-ethyl @ 2.0 L ha^-1^ (D_2_ × W_4_). The highest result of cob yield with husk was statistically similar with planted on 14 January with the application of herbicide pendimethalin @ 2.5 L ha^-1^ with one HW at 30 DAS (D_3_ × W_2_) and 15 December with two HW at 15 and 30 DAS (D_2_ × W_1_). The least cob yield with husk (0.88 t ha^-1^) and cob yield without husk (0.25 t ha^-1^) were found without weeded treatment planted in November (D_1_ × W_0_) and December (D_2_ × W_0_).

### Variation of fodder yield when planting at different times using different weeding management strategies

Fodder yield also showed variation under different weeding strategies and planting dates (Fig 2C). The maximum result (37.7 t ha^-1^) was recorded on 15 November planting with the application of herbicide pendimethalin @ 2.5 L ha^-1^ with one HW at 30 DAS (D_1_ × W_2_) and similar result was found in applying pyrazosulfuran-ethyl @ 2.0 L ha^-1^ with one HW at 30 DAS (D_1_ × W_3_) under same planting date. The least one (12.72 t ha^-1^) was measured from December planted without weeded plots (D_2_ × W_0_).

### Nutritional qualities of baby corn and fodder under different planting times and weeding regimes

#### Protein content (%)

On the protein content of baby corn, there is a substantial difference in the interaction between the date of planting and the weeding schedule. The highest protein content (20.20%) was recorded in plants sown on 15 December with the application of post-emergence herbicide with one HW at 30 DAS (D_2_ × W_3_) which is statistically similar to 15 November planting with two HW at 15 DAS and 30 DAS (D_1_ × W_1_) and the lowest result (8.73%) was found on 15 December planted plots with no weeding (D_2_ × W_0_) (Table 4). In the case of baby corn fodder, the protein content varies between leaf and stem. The highest protein content of leaf (18.70%) was recorded with the combination of 15 December planting with no weeding (D_2_ × W_0_) while the lowest one (6.00%) was found with 15 November planting with no weeding (D_1_ × W_0_) (Table 5). In stem, the maximum amount of protein content (4.90%) was obtained in both planted on 15 November with the application of pyrazosulfuran-ethyl @ 2.0 L ha^-1^ with one HW at 30 DAS (D_1_ × W_3_) and 14 January with two HW at 15 DAS and 30 DAS (D_3_ × W_1_) whereas the minimum amount was (2.40%) in 15 December planting with applying herbicide pendimethalin @ 2.5 L ha^-1^ followed by one HW at 30 DAS (D_2_ × W_2_) (Table 5).

**Table 4:** Effect of interaction between date of sowing and weeding regime on proximate components of baby corn.

Planting dates × weeding		Protein (%)	Carbohydrate (%)	Fat (%)	Fiber (%)	Ash (%)	Moisture (%)
15 November (D_1_)	W_0_	9.8 ± 1.31 i	50.4 ± 8.01 c	7.55 ± 1.58 j	11.4 ± 1.79 d	6.34 ± 1.18 d	11.59 ± 2.36 bc
	W_1_	20.2 ± 3.08 a	44.88 ± 6.41 f	8.25 ± 1.24 g	9.65 ± 1.32 e	5.36 ± 0.87 j	9.36 ± 1.78 h
	W_2_	19.53 ± 2.96 b	48.37 ± 8.82 e	6.95 ± 1.05 l	8.52 ± 1.19 k	5.46 ± 0.83 h	9.55 ± 1.61 g
	W_3_	19.53 ± 3.31 b	49.46 ± 6.61 d	7.38 ± 1.27 k	7.65 ± 1.41 m	6.51 ± 1.32 c	9.64 ± 1.65 g
	W_4_	18.1 ± 3.43 c	48.77 ± 10.57 e	8.25 ± 1.1 g	9.22 ± 2.01 f	5.84 ± 0.78 f	9.69 ± 1.3 fg
15 December (D_2_)	W_0_	8.73 ± 1.61 k	52.32 ± 8.97 a	7.71 ± 1.17 i	11.42 ± 2.08 cd	5.37 ± 0.75 ij	10.89 ± 2.01 d
	W_1_	9.1 ± 1.68 j	52.88 ± 7.21 a	9.05 ± 1.53 f	12.16 ± 2.55 b	7.13 ± 1.49 a	8.25 ± 1.25 j
	W_2_	17.3 ± 3.77 e	51.45 ± 9.37 b	7.41 ± 1.06 k	8.73 ± 1.9 j	6.21 ± 0.94 e	8.93 ± 1.54 3i
	W_3_	20.2 ± 3.26 a	43.57 ± 9.52 g	7.8 ± 1.69 h	12.7 ± 2.02 a	6.6 ± 1 b	9.65 ± 1.29 g
	W_4_	17 ± 2.24 f	43.69 ± 8.16 g	8.31 ± 1.69 g	11.47 ± 2.51 c	7.1 ± 1.23 a	9.84 ± 1.5 f
14 January (D_3_)	W_0_	10.5 ± 1.81 h	44.37 ± 7.18 f	10.63 ± 2.02 e	8.87 ± 1.34 i	5.14 ± 0.69 l	10.14 ± 1.72 e
	W_1_	18.1 ± 2.58 c	41.5 ± 6.32 i	11.62 ± 1.96 d	9.05 ± 1.55 g	5.41 ± 0.82 i	11.49 ± 1.64 c
	W_2_	17.7 ± 2.98 d	40.52 ± 8.25 j	13.24 ± 2.27 b	8.49 ± 1.16 k	4.97 ± 0.84 m	11.73 ± 2.54 3b
	W_3_	17.7 ± 2.78 d	40.35 ± 5.42 j	13.67 ± 1.84 a	8.96 ± 1.63 h	5.27 ± 0.75 k	12.26 ± 2.5 a
	W_4_	15.9 ± 3.47 g	42.35 ± 5.9 h	11.95 ± 2.2 c	8.22 ± 1.8 l	5.56 ± 1.21 g	12.28 ± 2.33 8a
Significance level		***	***	***	***	***	***

In a column, figures with same letter (s) or without do not differ significantly whereas figures with dissimilar letter differ significantly (as per DMRT), *** = Significant at 0.1% level of probability. Here, W_0_ = No weeding, W_1_ = Two hand weeding at 15 days after sowing (DAS) and 30 DAS, W_2_ = Pre-emergence herbicide (Pendimethalin) followed by one hand weeding at 30 DAS, W_3_ = Post-emergence herbicide (Pyrazosulfuran-ethyl) followed by one hand weeding at 30 DAS, W_4_ = Pre-emergence herbicide (Pendimethalin) followed by Post-emergence herbicide (Pyrazosulfuran-ethyl).

**Table 5:** Effect of interaction between date of sowing and weeding regime on proximate components of baby corn fodder.

Planting dates × weeding		Protein (%)		Carbohydrate (%)		Fat (%)		Fiber (%)		Ash (%)		Moisture (%)	
		Leaf	Stem	Leaf	Stem	Leaf	Stem	Leaf	Stem	Leaf	Stem	Leaf	Stem
15 November (D_1_)	W_0_	6.00 l	3.10d	36.36c	37.59d	4.16d	8.36h	28.85i	34.27i	5.75n	4.47b	18.93b	11.25h
	W_1_	12.40j	4.90a	39.44b	40.52b	2.69j	9.21 b	28.78i	28.17m	5.91m	3.72f	19.59a	11.72g
	W_2_	18.10b	3.50c	27.56 k	35.63f	3.27g	8.25 j	30.41h	33.17l	6.12 l	4.23c	13.63c	13.24 c
	W_3_	16.50d	4.90a	29.45hi	32.49i	4.17d	7.78 l	27.32 j	38.16d	7.22j	2.97i	12.25g	12.77e
	W_4_	17.40c	2.80d	31.53f	37.31d	2.87h	8.65 f	26.13k	33.80k	8.14d	3.55g	12.27g	12.72ef
15 December (D_2_)	W_0_	18.70a	4.20b	29.50h	36.54e	4.36 c	8.61g	25.75l	34.05j	7.13k	3.06h	13.24d	12.50 f
	W_1_	15.90e	3.47c	45.36a	35.45f	2.78 i	8.27i	34.48a	38.87c	8.41c	2.34 k	12.770f	11.20hi
	W_2_	13.60i	2.40e	33.29 e	39.45c	2.65 j	8.04k	32.17ef	35.12 g	8.59b	3.80e	8.47k	9.12k
	W_3_	17.40c	3.50c	35.37d	42.45a	1.88l	8.86 d	32.31de	38.12d	7.17k	2.37k	8.95j	10.74j
	W_4_	14.90g	3.50c	39.73b	34.41g	2.56 k	9.31a	21.76m	39.08b	7.84e	2.67j	12.39g	10.98ij
14 January (D_3_)	W_0_	7.60 k	3.50c	30.52g	30.69 j	3.62 f	8.85 d	31.27g	38.12d	7.66f	3.02hi	13.03e	14.02a
	W_1_	16.30d	4.90a	28.91i	34.48g	4.15d	7.05m	33.29c	35.74f	7.35i	3.91d	12.59f	12.92de
	W_2_	14.20h	4.20b	29.66h	33.56h	3.87e	8.88c	31.90f	34.80h	7.58g	4.23c	12.34g	13.70b
	W_3_	15.60f	3.47c	25.56l	29.49k	4.91b	8.78e	34.09b	39.69a	8.92a	5.69a	12.00h	13.17cd
	W_4_	14.20 h	3.50c	28.29j	30.33j	6.45a	8.88c	32.54d	37.63e	7.50 h	5.73a	11.64i	13.59b
Significance level		***	***	***	***	***	***	***	***	***	***	***	***
SE (±)		0.13	0.15	0.153	0.14	0.037	0.0065	0.14	0.031	0.0211	0.023	0.054	0.072

In a column, figures with same letter (s) or without do not differ significantly whereas figures with dissimilar letter differ significantly (as per DMRT), * = Significant at 5% level of probability, ** = Significant at 1% level of probability, *** = Significant at 0.1% level of probability. Here, W_0_ = No weeding, W_1_ = Two hand weeding at 15 days after sowing (DAS) and 30 DAS, W_2_ = Pre-emergence herbicide (Pendimethalin) followed by one hand weeding at 30 DAS, W_3_ = Post-emergence herbicide (Pyrazosulfuran-ethyl) followed by one hand weeding at 30 DAS, W_4_ = Pre-emergence herbicide (Pendimethalin) followed by Post-emergence herbicide (Pyrazosulfuran-ethyl).

#### Carbohydrate content (%)

The maximum quantity (52.88%) was obtained when planted on 15 December with two HW at 15 DAS and 30 DAS (D_2_ × W_1_) which is par with planted on 15 December with no weeding (D_2_ × W_0_) while the lowest amount (40.35%) was found in 14 January planting with application of pyrazosulfuran-ethyl @ 2.0 L ha^-1^ followed by one HW at 30 DAS (D_3_ × W_3_) (Table 4). The highest amount of carbohydrate content of leaf and stem of baby corn fodder (45.36%) and (42.45%) were recorded when sown on 15 December with two HW at 15 DAS and 30 DAS (D_2_ × W_1_) and in the application of pyrazosulfuran-ethyl @ 2.0 L ha^-1^ followed by one HW at 30 DAS (D_2_ × W_3_). The least amount (25.56%) and (29.49%) were obtained on 14 January planted using pyrazosulfuran-ethyl @ 2.0 L ha^-1^ with one HW at 30 DAS (D_3_ × W_3_) (Table 5).

#### Fat content (%)

Weeding and sowing dates have a considerable impact on the variation in fat content in baby corn and fodder. The highest fat in baby corn was (13.67%) estimated from 14 January with applying herbicide pyrazosulfuran-ethyl @ 2.0 L ha^-1^ with one HW at 30 DAS (D_3_ × W_3_) and the lowest content was (6.95%) from 15 November with the application of herbicide pendimethalin @ 2.5 L ha^-1^ followed by one HW at 30 DAS (D_1_ × W_2_) (Table 4). In the case of baby corn fodder, the highest fat content of leaf (6.45%) was obtained on 14 January sowing with the application of herbicide pendimethalin @ 2.5 L ha^-1^ followed by pyrazosulfuran-ethyl @ 2.0 L ha^-1^ (D_3_ × W_4_) and the lowest one was (1.88%) with 15 December with the application of pyrazosulfuran-ethyl @ 2.0 L ha^-1^ followed by one HW at 30 DAS (D_2_ × W_3_). For stem the maximum fat content was (9.31%) on 15 December sowing with the application of herbicide pendimethalin @ 2.5 L ha^-1^ and pyrazosulfuran-ethyl @ 2.0 L ha^-1^ (D_2_ × W_4_) and the minimum result was (7.05%) shown on 14 January planting with two HW at 15 DAS and 30 DAS (D_3_ × W_1_) (Table 5).

#### Fiber content (%)

Fiber content in baby corn and baby corn fodder varies significantly by sowing times and weeding strategies. The highest amount of fiber (12.70%) was found in 15 December planting using pyrazosulfuran-ethyl @ 2.0 L ha^-1^ along with one HW at 30 DAS (D_2_ × W_3_) and the lowest result (7.65%) was obtained under similar weeding regime in 15 November sowing baby corn (D_1_ × W_3_) (Table 4). The topmost amount of fiber on the leaf of baby corn fodder (34.48%) was recorded on 15 December planting with two HW at 15 DAS and 30 DAS (D_2_ × W_1_) and the lowest result (21.76%) was obtained with 15 December sowing applying both pre and pyrazosulfuran-ethyl @ 2.0 L ha^-1^ (D_2_ × W_4_) (Table 6). In stem, the maximum fiber was (39.69%) found on 14 January with the application of pyrazosulfuran-ethyl @ 2.0 L ha^-1^ followed by one HW at 30 DAS (D_3_ × W_3_) while the lowest one (28.17%) was resulted on 15 November with two HW at 15 DAS and 30 DAS (D_1_ × W_1_) (Table 5).

**Table 6:** Effect of interaction between date of sowing and weeding regime on mineral components of baby corn fodder.

Planting dates × weeding		Calcium (%)		Phosphorus (%)		Potassium (%)		Sulphur (%)	
		Leaf	Stem	Leaf	Stem	Leaf	Stem	Leaf	Stem
15 November (D_1_)	W_0_	0.01 n	0.010 m	0.194 f	0.142 n	0.077 n	0.097 k	0.05 j	0.09 b
	W_1_	0.04 k	0.026 i	0.327 de	0.135 o	0.081 m	0.097 k	0.04 l	0.06 d
	W_2_	0.05 j	0.020 l	0.311 e	0.299 a	0.113 g	0.988 j	0.01 n	0.07 c
	W_3_	0.06 i	0.050 c	0.341 c-e	0.215 h	0.101 i	0.149 b	0.07 d	0.09 b
	W_4_	0.06 h	0.060 b	0.408 b	0.293 c	0.121 f	0.145 c	0.05 g	0.04 e
15 December (D_2_)	W_0_	0.04 l	0.040 e	0.391 bc	0.241 f	0.089 l	0.149 b	0.02 m	0.07 c
	W_1_	0.03 m	0.040 d	0.334 de	0.185 i	0.097 k	0.124g	0.05 i	0.04 f
	W_2_	0.17 d	0.030 h	0.413 b	0.289 d	0.089 l	0.123 i	0.07 c	0.03 f-h
	W_3_	0.17 d	0.020 k	0.369 b-d	0.173 k	0.174 a	0.135 f	0.07 b	0.03 fg
	W_4_	0.20 c	0.020 j	0.552 a	0.228 g	0.129 d	0.153 a	0.06 f	0.02 h
14 January (D_3_)	W_0_	0.29 b	0.030 g	0.377 b-d	0.252 e	0.109 h	0.077 l	0.09 a	0.13 a
	W_1_	0.16 e	0.070 a	0.164 fg	0.296 b	0.134 c	0.123 h	0.07 e	0.05 de
	W_2_	0.36 a	0.040 f	0.135 g	0.168 m	0.100 j	0.142 e	0.07 e	0.02 h
	W_3_	0.14 f	0.040 d	0.158 fg	0.172 l	0.123 e	0.143 d	0.05 h	0.02gh
	W_4_	0.13 g	0.050 c	0.296 e	0.182 j	0.143 b	-	0.05 k	0.03 f
Significance level		***	***	***	***	***	***	***	***
SE (±)		0.00047	0.00018	0.00026	0.00020	0.00020	0.00013	0.00087	0.0070

In a column, figures with same letter (s) or without do not differ significantly whereas figures with dissimilar letter differ significantly (as per DMRT*** = Significant at 0.1% level of probability. Here, W_0_ = No weeding, W_1_ = Two hand weeding at 15 days after sowing (DAS) and 30 DAS, W_2_ = Pre-emergence herbicide (Pendimethalin) followed by one hand weeding at 30 DAS, W_3_ = Post-emergence herbicide (Pyrazosulfuran-ethyl) followed by one hand weeding at 30 DAS, W_4_ = Pre-emergence herbicide (Pendimethalin) followed by Post-emergence herbicide (Pyrazosulfuran-ethyl).

#### Ash content (%)

The amount of ash in baby corn and fodder was considerably impacted under different planting dates and weeding practices. The highest amount of ash (7.13%) was recorded with the combination of shown on 15 December with two HW at 15 DAS and 30 DAS (D_2_ × W_1_) treatments while the lowest amount of ash content (4.97%) was obtained on 14 January with the application of herbicide pendimethalin @ 2.5 L ha^-1^ followed by one HW at 30 DAS (D_3_ × W_2_) (Table 4). In the case of fodder, the ash content of leaf was recorded highest (8.92%) as shown on 14 January with the application of pyrazosulfuran-ethyl @ 2.0 L ha^-1^ followed by one HW at 30 DAS (D_3_ × W_3_) and the lowest result was (5.75%) found on 15 November with no weeding (D_1_ × W_0_) (Table 6). For stem, the maximum result (5.73%) was obtained with 14 January sowing using herbicide pendimethalin @ 2.5 L ha^-1^ and pyrazosulfuran-ethyl @ 2.0 L ha^-1^ (D_3_ × W_4_) followed by 14 January planting with application of pyrazosulfuran-ethyl @ 2.0 L ha^-1^ followed by one HW at 30 DAS (D_3_ × W_3_) and the lowest one (2.34%) was on 15 December planting with two HW at 15 DAS and 30 DAS (D_2_ × W_1_) (Table 5).

#### Moisture content (%)

Additionally, corn and fodder have different moisture contents. The highest amount of moisture content (12.28%) was recorded with the combination of shown on 14 January sowing with the application of herbicide pendimethalin @ 2.5 L ha^-1^ followed by pyrazosulfuran-ethyl @ 2.0 L ha^-1^ (D_3_ × W_4_) followed by in shown on 14 January with the application of pyrazosulfuran-ethyl @ 2.0 L ha^-1^ followed by one HW at 30 DAS (D_3_ × W_3_) and the lowest result (8.25%) was shown on 15 December with two HW at 15 DAS and 30 DAS (D_2_ × W_1_) (Table 4). In fodder, the highest moisture content on leaf (19.59%) and stem (14.02%) were obtained in the combination of shown on 15 November with two HW at 15 DAS and 30 DAS (D_1_ × W_1_) and shown on 14 January with no weeding (D_3_ × W_0_) while the lowest moisture content on leaf (8.47%) and stem (9.12%) were recorded with shown on 15 December with application of herbicide pendimethalin @ 2.5 L ha^-1^ followed by one HW at 30 DAS (D_2_ × W_2_) (Table 5).

### Mineral nutrient content of fodder under different planting times and weeding regimes

#### Calcium content (%)

Ca contents of stem and leaf of fodder were detected resulting in leaves having a substantially greater amount of Ca than stems (Table 6). The maximum Ca content on the leaf (0.36%) and stem (0.07%) was recorded with the combination of shown on 14 January with the application of herbicide pendimethalin @ 2.5 L ha^-1^ followed by one HW at 30 DAS (D_3_ × W_2_) and shown on 14 January with two HW at 15 DAS and 30 DAS (D_3_ × W_1_) with same planting time and the lowest result (0.01%) for both leaf and stem were found with the combination of 15 November planting with no weeding (D_1_ × W_0_).

#### Phosphorus (P) content (%)

The P content of fodder is greatly impacted by the combination of planting date and weeding practices (Table 6). The highest P content of leaf (0.552%) was obtained with 15 December sowing with the application of herbicide pendimethalin @ 2.5 L ha^-1^ followed by pyrazosulfuran-ethyl @ 2.0 L ha^-1^ (D_2_ × W_4_) and the lowest content (0.135%) was with the combination of shown on 14 January with application of herbicide pendimethalin @ 2.5 L ha^-1^ followed by one HW at 30 DAS (D_3_ × W_2_). The highest content of stem (0.299%) was recorded in shown on 15 November with the application of herbicide pendimethalin @ 2.5 L ha^-1^ followed by one HW at 30 DAS (D_1_ × W_2_) while the lowest one (0.135%) was found in shown on 15 November with two HW at 15 DAS and 30 DAS (D_1_ × W_1_).

#### Potassium (K) content (%)

The interaction effect of planting times and weeding affected K concentration in the leaves and stems (Table 6). The topmost findings of leaf K content (0.174%) were recorded in shown on 15 December with the application of pyrazosulfuran-ethyl @ 2.0 L ha^-1^ followed by one HW at 30 DAS (D_2_ × W_3_) whereas the lowest one (0.077%) was found in shown on 15 November with no weeding (D_1_ × W_0_). For the stem, the best output (0.153%) resulted in 15 December sowing with the application of herbicide pendimethalin @ 2.5 L ha^-1^ followed by pyrazosulfuran-ethyl @ 2.0 L ha^-1^ (D_2_ × W_4_), and the lowest one (0.077%) was found in shown on 14 January with no weeding (D_3_ × W_0_).

#### Sulphur (S) content (%)

The S content varied in the stems and leaves under the interaction effect of planting times and weeding regimes (Table 6). The plants with the greatest levels of S in their leaves (0.09%) and stem (0.13%) were found from 14 January with no weeding (D_3_ × W_0_). Whereas the least S content of leaf (0.01%) was recorded in shown on 15 November with the application of herbicide pendimethalin @ 2.5 L ha^-1^ followed by one HW at 30 DAS (D_1_ × W_2_) and stem (0.02%) with 15 December sowing with the application of herbicide pendimethalin @ 2.5 L ha^-1^ followed by pyrazosulfuran-ethyl @ 2.0 L ha^-1^ (D_2_ × W_4_) followed by the combination of shown on 14 January with the application of herbicide pendimethalin @ 2.5 L ha^-1^ followed by one HW at 30 DAS (D_3_ × W_2_).

## Discussion

For ensuring higher yield and quality of crops, suitable planting time and weeding schedule are crucial prerequisites. Weed infestation also depends on planting times (Table 2). The plots that were sown on November 15 and treated with herbicide pendimethalin @ 2.5 L ha^-1^, followed by one HW at 30 days after sowing had the lowest weed density. The lowest weed dry matter was found in December 15 sowing and treated an herbicide pendimethalin @ 2.5 L ha^-1^ followed by the pyrazosulfuran-ethyl @ 2.0 L ha^-1^ application. Baby corn, when sown at different times along with various weed management strategies displayed a notable difference in the cob and green fodder yield, and their nutritional quality (Tables 3–6 and Fig 2). The cooler temperatures and better moisture conditions in mid-December promote healthy seedling establishment and reduce weed pressures. The comparatively taller plants were found in December and January sowing with two HW at 15 and 30 DAS. Sharma et al. [31] also reported taller corn plants under two HW compared to other weeding methods. Plants produced the most cobs when planted early using pyrazosulfuran-ethyl @ 2.0 L ha^-1^ with one HW, whereas they showed a lower number of cobs under no weeding at similar planting time. Chopra and Angiras [32] documented that the post-emergence herbicide application increased the number of maize cobs in the plants.

Plants grown without weeding had a fierce crop-weed competition that led to lowering the yield (Fig 2). Densely populated weeds enhanced crop-weed competition and reduced the number of cobs plant^-1^. The plants treated with pre and post emergence herbicides were found to have the greatest cob diameter. Due to the higher crop-weed competition in the plots, the cob weight without husk was the lowest in the plants that had not been weeded. Mandal et al. [33] observed that pre-emergence and post-emergence herbicides application increased maximum cob weight. Variations in the cob production at different seasons might be caused by the effective use of moisture by crops at the ideal planting date, when the moisture remained in the soil profile during the winter months. The cobs from the plants that were sown on December 15 were the longest and greatest in size (Table 3), they yielded the maximum corn without husk (Fig 2A). By applying herbicide pendimethalin @ 2.5 L ha^-1^ and pyrazosulfuran-ethyl @ 2.0 L ha^-1^ and sowing in December showed better cob yield as reported by Patel et al. [34]. Furthermore, a later sowing date increases soil moisture content during the critical seed-filling phase which would lower cob yield performance.

November sowing with two-HW at 15 and 30 DAS produced the highest amount of fodder and 15 December planting without weeding treatment gave the least quantity of fodder (Fig 2C). There was a propensity for the yield to decrease as the planting season progressed. This might be the result of varied meteorological parameters during the growing season as a result of varying sowing dates. The capacity of the crop to efficiently use moisture at earlier sowing resulted in more efficient vegetative development up to harvest than at later sowing. A similar pattern was also described by Shaheenuzzamn et al. [35].

The times of planting and the frequencies of weeding had a considerable impact on the quality of baby corn and green fodder (Tables 4–6). Proteins in cob are augmented by the control using of herbicides and HW. In fodder, the protein content of leaves was greater than that of stems, with the greatest value for leaves being recorded in December sowing without weeded plants while the highest protein values for stems were reported in two-hand weeded plants in both early and late sown corn (Table 5). Comprehensive weed control ensures minimal competition for resources, resulting in higher yields and improved nutritional quality of the cobs.

In general, a late planting date can readily impair the grain quality of all corn. Protein is an essential quality component and a source of nutrient dense feed for both human and animal nutrition as well as its use in many other industries. Planting dates had a significant influence on varying amounts of protein. Early planting significantly increased protein content while late sowing significantly decreased it [36]. This seems to be caused by the emergence of maturity phases, a decline in the leaf to stem ratio of the plant and hardening of the stems as the culture period increases. The acid detergent fiber and total digestible nutrients did not show significant variation due to sowing dates. The chemical composition of fodder crops varied significantly depending on the time of cutting and the date of sowing [37].

The combination of planting and weeding practices had a significant influence on the Ca, P, K, and S content of leaves and stems (Table 6). The Ca, P and S content of the leaves and stems increased by applying herbicides and HW and reduced when no weeding was performed. The mineral elements in forage are important because they are essential to animal metabolism and body cell function. It turns out that ash forage is a reliable predictor of the amount of minerals in plant tissue [38]. The findings of this study demonstrated a significant relationship between the weeding strategies and planting times. Our research findings suggested that the application of herbicidal weed control or herbicide along with HW and sown at suitable times might be beneficial for enhancing baby corn and fodder yield and quality performance. It is important to schedule crop management methods properly since the highest nutrient concentrations can be achieved.

## Conclusion

The findings of this experiment conclusively showed that the timing of the sowing and weeding schedule has a major impact on the quantity and quality of baby corn and green fodder. It would be concluded that planting baby corn on 15 December and using pendimethalin @ 2.5 L ha^-1^ (pre-emergence) followed by pyrazosulfuran-ethyl @ 2.0 L (post-emergence) herbicides resulted in the maximum cob yield with husk (11.93 t ha^-1^) and cob yield without husk (3.07 t ha^-1^) levels and the highest protein level (20.20%) was recorded in same date of sowing with pyrazosulfuran-ethyl + one HW at 30 DAS.

## Supporting information

S1 Fig Experimental field layout. (TIFF)
